# Promoting Self-Regulation in Children with Cerebral Palsy: A Mixed Analysis of the Impact of a Training Program for Psychologists

**DOI:** 10.3390/ejihpe15070120

**Published:** 2025-06-26

**Authors:** André Oliveira, Inês Castro, Ana Guimarães, Sofia Vidal, Maria Carneiro, Beatriz Magalhães, Pedro Rosário, Armanda Pereira

**Affiliations:** 1 Department of Psychology and Education, Faculty of Social Sciences and Humanities, University of Beira Interior, 6200-209 Covilhã, Portugal; andre.soares.oliveira@ubi.pt; 2 Psychology Research Center, School of Psychology, University of Minho, 4710-057 Braga, Portugal; inesrodcastro@gmail.com (I.C.); anacunha1296@gmail.com (A.G.); sip.vidal@gmail.com (S.V.); mariahcarneiro11@gmail.com (M.C.); prosario@psi.uminho.pt (P.R.); 3 Department of Education and Psychology, School of Human and Social Sciences, University of Trás-os-Montes and Alto Douro, 5000-801 Vila Real, Portugal; beatrizmagalhaes100@gmail.com

**Keywords:** cerebral palsy, self-regulation, narrative-based intervention, training program, psychologists, sequential explanatory mixed-methods design

## Abstract

Cerebral palsy is a neurodevelopmental disorder that can impair self-regulatory skills. Narrative-based tools, such as “The Incredible Adventures of Anastácio, the Explorer”, have shown promise in fostering these competencies in children with cerebral palsy. This study evaluated a training program for psychologists using a story-based intervention to promote self-regulation skills in children with cerebral palsy. A sequential explanatory mixed-methods design assessed declarative and procedural knowledge and self-efficacy changes. Seventeen psychologists completed a three-level program: Level 1 included sociodemographic and declarative knowledge assessments (pre/post); Level 2 focused on self-efficacy (pre/post) and a procedural knowledge task; and Level 3 reassessed self-efficacy (post 2). Semi-structured interviews explored participants’ experiences qualitatively. Results showed a significant increase in declarative knowledge, confirming the effectiveness of training. All participants demonstrated positive levels of procedural knowledge despite individual variations. Self-efficacy did not increase significantly, likely due to the tool’s high initial score and novelty. Qualitative findings highlighted the value of balancing theoretical and practical components in training. Although self-efficacy gains were limited, the program enhanced psychologists’ declarative and procedural knowledge. Findings suggest that narrative-based training may help professionals update their knowledge and practices, supporting the promotion of self-regulatory skills in children with cerebral palsy.

## 1. Introduction

Cerebral palsy (CP) is a neurodevelopmental disorder that occurs in early childhood and persists throughout life (Rosenbaum et al., 2007). It affects approximately 1.5 per 1000 live births in Europe and 1.3 per 1000 in Portugal (McIntyre et al., 2022). The CP term encompasses a group of permanent but non-progressive disorders that affect movement and posture (Aisen et al., 2011; Rosenbaum et al., 2007), and the primary symptoms (i.e., muscle tone, strength, coordination, and movement changes) accompanied by secondary impairments related to sensation, perception, behaviour, communication, cognition, musculoskeletal issues, and epilepsy (Rosenbaum et al., 2007). Secondary impairments will likely develop over time and may become functional through interventions (Jeffries et al., 2016). Among these challenges, attention and executive function, while critical for all children, are often impaired in children with CP due to underlying brain injury (Stadskleiv et al., 2018; Tayyare et al., 2023). These impairments can significantly impact this population’s regulation of behaviours and emotions, directly undermining self-regulation (SR) capacities–essential for initiating, sustaining, and guiding thoughts, emotions, and behaviours toward learning goals (Stadskleiv et al., 2018). Therefore, SR competency training is crucial in children with CP. This helps reduce the impact of impairments and promotes autonomy. Early introduction of SR training interventions benefits from the neuroplasticity potential of young children (Graham et al., 2016). While SR promotion interventions for children with CP have demonstrated to be efficacious (e.g., Oliveira et al., 2024; Pereira et al., 2019), many existing programmes predominantly focus on motor or cognitive rehabilitation, neglecting essential psychological and behavioural components crucial for autonomy and participation (Novak et al., 2013, 2020; Wade, 2016, 2020).

Furthermore, there is a paucity of structured, evidence-based programmes specifically designed to train psychologists in implementing adapted SR interventions for this clinical population. Psychologists play a pivotal role in supporting children with CP, as they are equipped to address cognitive and emotional challenges and adapt strategies to individual needs and family contexts. However, without targeted training, professionals may lack the specific competencies required to effectively implement innovative tools (Wade, 2020). Despite growing recognition of SR’s importance for children with CP, literature exploring the impact of narrative-based interventions delivered by trained psychologists remains scarce. Most research has focused on direct child interventions, often overlooking the need to equip professionals with relevant knowledge and implementation skills. The present study aims to address this gap by evaluating a structured training programme for psychologists centred on a narrative tool specifically developed for children with CP.

Narrative-based interventions are innovative as they provide meaningful and engaging contexts that foster intrinsic motivation and enable children to learn through observation via identifiable characters and stories (Rosário et al., 2016b, 2017). Compared to traditional approaches, such as behavioural methods, narratives may enhance engagement, reflection, and the transfer of SR strategies to real-life situations, demonstrating potential superiority for the CP population. Narrative-based interventions effectively facilitate children’s learning of SR strategies and autonomy development (e.g., Rosário et al., 2016b). Narrative structure is a valuable tool for guiding children to understand SR aspects and reflect on how to apply these strategies in their lives. This intervention model aims to create a supportive environment to facilitate discussions on the various difficulties children with developmental disorders may face (Pereira et al., 2019). Efforts to develop children’s proximity to the narrative help them actively engage in the learning process while facilitating discussion with peers and fostering a positive attitude towards the strategic content of the narrative. The literature indicates that students’ motivational profiles significantly affect their engagement with and the efficacy of interventions designed to enhance self-regulation skills. Learning-oriented motivation is associated with higher levels of engagement, greater control over the learning process, and reduced anxiety (Valle et al., 2010).

Furthermore, narrative-based interventions enable readers to explore different perspectives on approaching new challenges and problem-solving. By modelling characters’ behaviours, children can also learn how to self-regulate their behaviour, thoughts, and feelings (Rosário et al., 2017). Discussing characters’ actions in the plot helps children develop new behaviours and learn vicariously through social modelling, an approach supported by recent research on observational learning and self-regulation in educational settings (Schunk & DiBenedetto, 2020) The story-tool “The Incredible Adventures of Anastácio, the Explorer” (Rosário et al., 2016a) was designed to enhance SR competencies and strategies in children with developmental disorders, such as CP. This narrative tells the story of a boy named Anastácio, who goes on an adventure in his home (https://anastacio-projecto.weebly.com; accessed on 20 June 2025). In the narrative plot, Anastácio encounters numerous challenges and obstacles during his journey, all while remaining steadfast in his primary objective of locating a plant capable of curing rabbits. Integrated within the story plot are SR strategies that play a critical role in guiding the protagonist through adversities. Children with CP can read the story, engage in discussions about its content, and use intricately connected SR strategies (Pereira et al., 2019). The story emphasises that goals can be achieved with effort and persistence, with children learning how SR strategies could be fundamental to help them face challenges and achieve their goals. These strategies are based on the cyclical PLEE model (i.e., planning, execution, and evaluation; Rosário et al., 2017), amplifying the cyclical nature of Zimmerman’s self-regulated learning model, structured in three phases: forethought, performance, and self-reflection (Zimmerman & Labuhn, 2012). Previous data show a positive impact of training with this story tool on promoting SR in children with CP. Specifically, the findings indicate that children who underwent this intervention improved their SR competencies and ability to identify and apply them in their daily lives, particularly in educational (Pereira et al., 2019) and rehabilitation (Oliveira et al., 2024) contexts. Trained educational psychology researchers conducted this intervention program in the scope of the research projects. However, to fully harness its benefits, this tool must be readily accessible to those who interact with children with CP daily, such as psychologists in rehabilitation centres. In addition, these professionals need training on SR competencies and intervention tools that are likely to meet these children’s educational needs and provide effective support (Wade, 2020). Therefore, the overarching objective of the current mixed-method study was to develop and evaluate a structured training program designed to equip psychologists with the necessary theoretical and practical knowledge to effectively implement and assess an SR intervention program based on the story-tool “The Incredible Adventures of Anastácio, the Explorer” (Rosário et al., 2016a).

Discussing the storyline and encouraging children to reflect on their behaviour to promote SR competencies requires expertise. Additionally, practice sessions with tasks related to the story and intervention goals are expected to stimulate the learned competencies. The ability of psychologists to engage children in reflecting on the narrative messages of a narrative-based SR intervention and its application in their daily lives could be one of the precipitating factors in achieving positive results with the intervention. For these reasons, psychologists must be well-prepared and theoretically intentional in their actions. Therefore, to implement this intervention, it could be necessary to involve training and support in understanding the narrative and developing effective approaches for the sessions. Professional training is essential to achieve optimal outcomes in any field. For example, in health and organizational settings, research has demonstrated that trained professionals consistently perform better than those who lack adequate training (e.g., Abogsesa & Kaushik, 2017; Hatfield et al., 2020). We hypothesised that it is equally valid for professionals who use specific tools or interventions, such as narrative-based intervention tools. When adequately trained, professionals are more likely to apply these tools correctly and achieve desired outcomes. Recognising the importance of providing effective tools to rehabilitation staff (Wade, 2020), specifically psychologists, and recognising the need for specific training to apply the tool effectively, we developed a training program for psychologists in CP rehabilitation centres. This training program focused on Anastácio’s story, ensuring that professionals could implement SR intervention effectively. With this training program, we aimed to enhance psychologists’ ability to provide effective support, thus promoting autonomy and improving the quality of life of individuals with CP.

Training psychologists in implementing a narrative-based intervention to promote SR is expected to provide psychologists with the ability to develop declarative, procedural, and conditional knowledge in a specific domain. Declarative knowledge describes both factual and descriptive information. The current program corresponds to learning theories that sustain the intervention. Procedural knowledge relates to using different strategies to address a given task, which in training refers to knowing how to achieve self-set intervention objectives. Finally, conditional knowledge is associated with the ability to perceive when to use a particular process (Moshman, 2018). This knowledge is key to understanding the advantages and constraints of specific strategies. Moshman (2018) mentioned that this knowledge is achieved only when professionals apply the tool in context. During training to implement a particular intervention tool, trainees are expected to gradually feel more competent (i.e., increase their perception of self-efficacy).

Self-efficacy refers to an individual’s belief in their ability to successfully perform behaviours required to achieve specific goals or outcomes, a construct empirically substantiated by contemporary systematic reviews in educational and psychological research (Honicke & Broadbent, 2016; Schunk & DiBenedetto, 2020). It evolves based on previous experiences and performance outcomes, which inform individuals about their (in)ability regarding a particular task or area of knowledge (Burke & Hutchins, 2007; Shukla et al., 2023). Professionals who receive adequate training in using specific tools or interventions tend to report higher self-efficacy in applying them, which is associated with more effective implementation and improved outcomes (Shapiro et al., 2021). Therefore, by investing in training and promoting self-efficacy, professionals can effectively enhance their ability to implement interventions using narrative tools. Consequently, professionals are prompted to promote the development of SR competencies in children with CP, which has been shown to positively impact academic (Pereira et al., 2019) and rehabilitation (Oliveira et al., 2024) outcomes.

### The Current Study

We have developed a training program to enable psychologists with theoretical and practical knowledge to implement and evaluate SR intervention based on the story-tool “The Incredible Adventures of Anastácio, the Explorer” (Rosário et al., 2016a). To this end, the training was organised into three levels, integrating the theoretical and practical components. Level 1 delivered the theoretical foundations of the program and focused on the fundamental theories that support the intervention program, while Levels 2 and 3 focused on analysing the story tool and training on how to apply for the program. Specifically, the training program aimed to improve psychologists’ declarative and procedural knowledge of the narrative-based intervention tool, theoretical background, and principles guiding its application. In this training, conditional knowledge was not assessed. Despite a brief discussion covering conditional knowledge, this knowledge facet will only be fully implemented when psychologists apply the tool. The first level of the training program was predominantly theoretical, providing a theoretical background for the intervention’s principles. Each session covered motivation, SR, and the narrative-based intervention (see Table 1). The second level introduces the theoretical-practical component. Participants received training on the book’s content, structure, and methodological tools required to conduct the intervention. Two of the main tools are interrelated: (i) training on questioning following four typologies, i.e., stimulating attention, constructing arguments, constructing divergent solutions, and decision-making, and (ii) the questioning process that guides the story content discussion is driven by three types of conceptual knowledge: declarative (what is goal setting?), procedural (How do you set a goal?), and conditional (When do you set a goal?). Following theoretical training, participants will be encouraged to engage in practical applications through tasks and exercises.

The third level of the training course was practical. Participants attended a session on the intervention tool to provide an actual implementation example. Afterward, the participants formulated a plan for an intervention session, culminating in a practical exercise to apply the acquired knowledge within a simulation task–the simulation task aimed to evaluate trainees’ proficiency (see Supplementary Materials). Three experts in narrative-based intervention were invited to assess their performance in groups of 2 or 3 individuals. An observation grid was crafted to facilitate this comprehensive assessment, aligned with the principles of the simulated learning theory and specific training objectives. The experts observed and noted the presence of specific behaviours in the trainees and assessed their quality. The criteria encompassed aspects such as “the implementer’s ability to maintain the children’s engagement”, “the implementer’s adept use of various questioning techniques to stimulate reflection”, and “the implementer’s capacity to validate and appreciate children’s contributions, even when they deviate from the anticipated responses.” This exercise is theoretically based on simulated learning, a methodology that creates a situation or environment that allows people to experience an approximation of an actual event to practice, learn, or evaluate (Paparo et al., 2021). This active learning methodology has several benefits, such as providing a structured environment where participants can practice skills without negative consequences. The expected result is better preparedness and an increased ability to respond to task demands (Paparo et al., 2021). The results of this training level are integrated into the discussion section.

Accordingly, we formulated the following research questions to guide our investigation: (1) What is the impact of level one content on the declarative knowledge (i.e., basic theoretical principles) of participants?; (2) What is the participants’ level of ability to apply theoretical knowledge (i.e., declarative knowledge, level 1) and practical knowledge (i.e., procedural knowledge, level 2)?; (3) What is the impact of training on psychologists’ perceived self-efficacy in implementing an SR intervention program? We hypothesised that training would increase participating psychologists’ declarative and procedural knowledge and self-efficacy. Given the qualitative methodology of the fourth research question, we do not propose any specific hypotheses.

This study followed a two-phase explanatory sequential mixed-method design to examine the impact of a training program in Anastácio’s narrative-based intervention. To address the difficulties in finding a common schedule to set the program, we developed two training editions following the same design (e.g., hours of training). The first edition occurred between November 2022 and July 2023, and the second between November 2023 and February 2024. In the first edition, psychologists from three different centres participated in the study. This edition was held in a hybrid format (i.e., online or in person). In the second edition, psychologists from three centres participated in the study. Given the distance between the centres participating in the second edition and the university, all sessions were conducted online.

## 2. Materials and Methods

This study followed a two-phase explanatory sequential mixed methods design to examine the impact of a training program in a narrative-based intervention, “The Incredibles Adventures of Anastácio, the Explorer”. To address the difficulties in finding a common schedule to set the program, we developed two training editions following the same design (e.g., hours of training). The first edition occurred between November 2022 and July 2023, and the second between November 2023 and February 2024. In the first edition, psychologists from three different centres participated in the study. This edition was held in a hybrid format, with all sessions conducted either online or in person. In the second edition, psychologists from three centres participated in the study. Given the distance between the centres participating in the second edition and the university, all sessions were conducted online.

### 2.1. Quantitative Phase Materials and Methods

#### 2.1.1. Sample

To recruit participants for the study, we contacted 15 Portuguese rehabilitation centres via email or telephone. Three of those (20%) did not respond. One rehabilitation centre informed us that the population did not fit the intended population. A meeting between the training team and the directors of the remaining centres (*n* = 11, 73.3%) was held to convey the objectives and structure of the training. As a result of these meetings, five centres informed the research team that they could not participate because of incompatible schedules and a lack of professionals working with the tool’s target audience. Therefore, 17 psychologists from six (40%) CP rehabilitation centres agreed to participate in the training. To participate in the training program, the participants in this study only had to be psychologists in the rehabilitation centre who were directly involved with children with CP. Psychologists whose scope of intervention focused only on adolescents or adults with CP were excluded. This exclusion criterion was implemented because the intervention instrument and training programme were specifically developed to target distinct developmental requirements and SR difficulties characteristic of paediatric populations with CP. The detailed sociodemographic data of the participants are presented in Table 2.

#### 2.1.2. Assessment Protocol and Procedure

**Figure 1:**
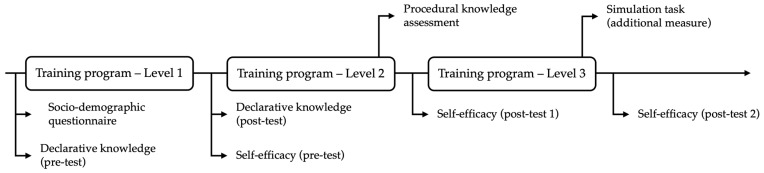
Partial (quantitative phase) research design.

Figure 1 illustrates the research design of the quantitative phase. After each of the three levels ended, the participants were invited to complete an assessment protocol to monitor their progress and learning. Before beginning Level 1, we administered a sociodemographic questionnaire. In addition, a pre-test was conducted to assess previous declarative knowledge (declarative knowledge pre-test). Post-test data were collected using the same questionnaire after completing all the sessions at this level. Before beginning Level 2, the first moment of the self-efficacy questionnaire (self-efficacy pre-test) was collected. The decision to conduct the pre-test before Level 2, rather than before Level 1, came from the recognition that assessing perceived competence requires some knowledge of the task. Learners require some familiarity with the subject matter to project themselves to perform the task, thus assessing their sense of capability. Before Level 1, participants lacked awareness of the tool and its implementation, making it possible that self-efficacy assessments would result in unrealistic results that are uninformed about reality. Post-tests were collected after completing Level 2 (self-efficacy post-test 1) and Level 3 (self-efficacy post-test 2). There was one moment to assess procedural knowledge during Level 2.

#### 2.1.3. Variables and Instruments

##### Socio-Demographic

To characterise the psychologist participants, we administered a questionnaire with 40 questions that collected information such as gender, age, and nationality. We also collected data on academic qualifications, years of experience as a psychologist, and years of experience as a psychologist in rehabilitation. Finally, we collected the approximate number of hours previous training participants enrolled in and the topics covered in that training.

##### Declarative Knowledge

A questionnaire was drawn up for this research to assess their declarative knowledge. Experts in the assessed topics made it and included 40 questions, 21 of which were ‘true or false’, and 19 were single choice. The questions referred to the theoretical principles covered in the Level 1 sessions. Some examples of true and false questions include: “The psychologist can motivate the client”, “Self-determination is considered a dispositional characteristic manifested as action as a causal agent in one’s life”, “Just reading stories is enough to promote the instruction and transfer of self-regulatory strategies.”

##### Procedural Knowledge

Psychologists were asked to perform a written simulation task to assess procedural knowledge. The written task was included in Level 2. Participants were asked to select one chapter of “The Incredible Adventures of Anastácio, the Explorer” (Rosário et al., 2016a) and then identify the intervention goal to guide the development of a questioning script. The evaluation followed the criteria defined by the research team based on the main goals of each training course level. Three research team members graded each task, and agreement between the researchers was calculated (*k* = 0.88).

##### Self-Efficacy

The Portuguese adaptation of the Generalised Self-Efficacy Scale (Nunes et al., 1999) was administered to assess the participants’ self-efficacy in the intervention program implementation. The participants were asked to fill in the scale items regarding their perceived competence in implementing Anastácio’s SR intervention program. The scale comprises ten questions (e.g., “I can always solve difficult problems if I try hard enough”, “It’s easy for me to stick to my intentions and achieve my goals”) and uses a 4-point Likert scale, in which participants indicate their degree of agreement with each item, ranging from “not at all true” to “exactly true”. This scale stands out for its explicit emphasis on personal agency, signifying that one’s actions can lead to successful outcomes, especially in stress-inducing situations (Leme et al., 2013). All items are worded positively, so high scores indicate the presence of high general self-efficacy. It is a unidimensional scale in which the ten items converge in assessing the global construct of self-efficacy (Araújo & Moura, 2013). The scale’s reliability in this study was highly satisfactory, as indicated by a Cronbach’s alpha coefficient of 0.87.

#### 2.1.4. Data Analysis

Statistical analyses were performed using IBM^®^ SPSS^®^ Statistics^TM^ software (version 29.0; IBM Corp, 2022) for Windows^®^. The analysis included (i) a descriptive analysis of the data, (ii) verification of the data normality using the Shapiro-Wilk test, (iii) a paired samples *t*-test to compare the results of declarative knowledge in the pre-test and post-test, (iv) for the procedural knowledge data, we performed a descriptive analysis using the percentiles (i.e., 25, 50 and 75), (v) verification of the assumption of sphericity of self-efficacy data, using Mauchly’s test of sphericity, and (vi) a Multivariate Analysis of Variance to analyse changes in self-efficacy over time.

### 2.2. Qualitative Phase Materials and Methods

#### 2.2.1. Sample

For participant selection, we analysed the differences between participants, and we found two distinct profiles of participants according to their results in the individual measures (i.e., all the evaluation measures except the simulation session). The assignment of profiles was determined by a combination of participants’ knowledge and self-efficacy scores, as follows: (i) higher knowledge but lower self-efficacy (profile A), and (ii) lower knowledge but higher self-efficacy (profile B). To do this, we sorted the results of knowledge measures (declarative and procedural knowledge measures added together) and self-efficacy measures in ascending order and assigned the participants who were simultaneously at the top of one measure and the bottom of the other (i.e., we considered the three best results and the three worst results as “top”). Thus, five participants were selected for the interviews, one of whom did not respond to the invitation. The remaining four samples were evenly divided between the profiles.

In profile A, the two participants had 11 and four years of experience as psychologists, and 10 and 4 years of experience in CP rehabilitation centres, respectively. Both participants had below-average years of experience. No participant reported prior training on the topics covered in the current training. Participant 1 (P1A) was part of a group in the simulation session with a score of 69.5%. The group in which P2A was enrolled scored 75%.

In profile B, the two participants had nine and 39 years of experience as psychologists and CP rehabilitation centres, respectively. Notably, participant 1 (P1B) had already received training in the topics covered in the current training and had experience with Anastácio’s story tool. P1B was part of a group in the simulation session with a score of 65.3%. The group in which P2B was enrolled scored 76.4%.

#### 2.2.2. Procedure

**Figure 2:**
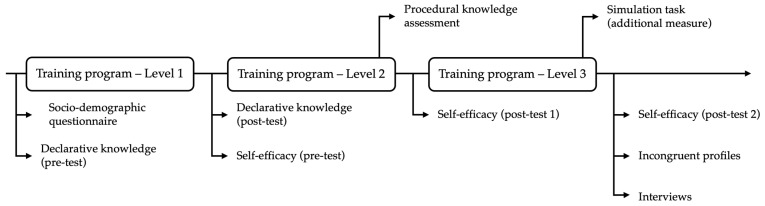
Full (quantitative + qualitative phases) research design.

Figure 2 illustrates the research design of the quantitative and qualitative phases. After determining which participants met the criteria, they were contacted via e-mail to complete an interview. The period between the last training session and the interview ranged from two to three weeks. The interviews were conducted using Zoom^®^ (Zoom Video Communications, 2011) videoconferencing software and lasted between 20 and 30 min.

#### 2.2.3. Interviews

Semi-structured interviews were conducted to identify training program topics considered crucial or less relevant for implementing the intervention tool. We also aimed to understand the factors contributing to the assessment outcomes. For the development of the interview script, we followed “The Self-System Model of Motivational Development” (Dincer et al., 2019; Skinner et al., 2008), which encompasses four motivational variables: context variables (i.e., related to the social environment), self-variables (i.e., beliefs and competence perceptions), action variables (i.e., goal-oriented behaviours), and outcome variables (i.e., learning development; Dincer et al., 2019). For instance, sample questions included: “What do you think about the training being carried out in a group?”; “What skills do you think you could develop or consolidate during the training?”; “How did you organize your preparation before each session?”; “If you had to implement the intervention program today, how would you feel?”.

#### 2.2.4. Data Analysis

First, interviews were transcribed verbatim. A thematic analysis approach was used to identify themes from the coded segments. In accordance with Braun and Clarke’s (2021) six-phase analytical framework, the initial stage following transcription involved (i) data familiarization. An initial codebook with general themes was drawn up: steps (ii) coding and (iii) searching for themes. Two researchers then coded the interview transcripts with the initial codebook while reviewing and adding codes to the codebook: steps (iv) reviewing themes and (v) defining and naming themes. This process ensured the codes adequately represented the significant information units conveyed during the interviews. Disagreements over the coding and revision of the codification led by the two researchers were discussed and resolved to reach a consensus (*k* = 0.9). The analysis and writing (step (vi) process was assisted by NVivo^®^ 14 software (QSR International, 2021) for Windows^®^.

## 3. Results and Findings

### 3.1. Quantitative Phase Results

#### 3.1.1. Declarative Knowledge

The results of the Shapiro-Wilk test for the pre-test data were *t* (17) = 0.947, *p* = 0.409, and the post-test *t* (17) = 0.938, *p* = 0.297, both following a normal distribution. On average, participants scored higher on the post-test of the declarative knowledge questionnaire (*M* = 69.059, *SE* = 1.149) than on the pre-test (*M* = 63.229, *SE* = 0.617), *t* (17) = −5.204, *p* < 0.001, data are statistically significant.

#### 3.1.2. Procedural Knowledge

The data indicated that participants showed positive results in the exercise (*M* = 67.79%, *SE* = 11.95%). The percentile analysis showed that 25% of the participants scored below 61.25%, 50% scored below 72.50%, and 75% of the students scored below 78.75%. The interquartile range, 21.25, indicates the dispersion of the results. These data suggest that participants’ results varied moderately, were congruent with declarative knowledge results, and that the exercise effectively captured the participants’ acquired knowledge application.

#### 3.1.3. Self-Efficacy

The results of the Shapiro-Wilk test for the pre-test data were *t*(17) = 0.940, *p* = 0.316, post-test 1 *t*(17) = 0.943, *p* = 0.358, and post-test 2 *t*(17) = 0.914, *p* = 0.116, all following a normal distribution. Mauchly’s test of sphericity was performed to assess the assumption of sphericity in the dataset. The data, χ^2^(2) = 1.373, *p* = 0.503, indicate that the assumption of sphericity was met. Using Wilks’ statistics, we found no significant change in participants’ self-efficacy during training, Λ = 0.929, *F*(2, 15) = 0.572, *p* = 0.576 (see Table 3). However, the descriptive statistics showed that, although not significant, there was a slight increase from pre-test (*M* = 3.377, *SE* = 0.419) to post-test 1 (*M* = 3.412, *SE* = 0.381) and post-test 2 (*M* = 3.447, *SE* = 0.318; see Table 4).

These results suggest that all participants performed satisfactorily in the evaluations and were adjusted to the training objectives (declarative knowledge post-test: *M* = 69.059, *SE* = 1.149; procedural knowledge post-test: *M* = 67.79, *SE* = 11.95; self-efficacy post-test 2: *M* = 3.447, *SE* = 0.318); however, we identified some profiles that deviated from the expected pattern. We expected that participants with greater knowledge of the tool would also demonstrate greater self-efficacy (Boswell, 2013; Constantine et al., 2019). However, when we ordered the results of these two variables, we found that some participants were at the top of one and the bottom of the other, an incongruous profile. Therefore, we decided to study these unexpected profiles to understand their contribution further. Hence, we posed a fourth qualitative research question: “What are the perceptions and experiences of psychologists with incongruent profiles (misfit between knowledge and self-efficacy) about the training to implement the intervention program?”

### 3.2. Qualitative Phase Findings

As presented in Table 5, three themes emerged from the data: (1) participants’ academic backgrounds and professional experiences, (2) features of the training model, and (3) dispositional profiles. The findings are presented according to the order of the identified themes. The participants’ verbatim quotes were introduced to illustrate the categories and patterns identified.

#### 3.2.1. Participants’ Academic Background and Professional Experience

Regarding the first theme, it was observed that participants in profile B frequently mentioned their academic and professional backgrounds. For example, their years of professional experience and how this made them feel able to apply the tool successfully, as Participant 1 of profile B (P1B) said when asked about the contribution of training to the application of the tool:

P1B: *[…] It contributed to the improvement; more than the training, I have the contribution of experience.*

However, the same participant mentioned a barrier to their professional experience.

P1B: *[…] Especially for those who have been working for a while and sometimes go a bit automatically. Going back to the theory to get information, sometimes, since everything is automatic, you always have to go back because sometimes the theoretical foundation is there, but you need to know how to dig deeply to find out what is really at the base.*

Compared with profile A, these participants referred more often to what they already knew and what they had acquired in their previous training.

P2B: *I am almost a year and a half away from retiring, and it has allowed me to remember concepts and deepen others that I did not have in my practice that were intuitive or acquired through theoretical curiosity.*

By intersecting with “The Self-System Model of Motivational Development” (Dincer et al., 2019; Skinner et al., 2008), it is possible to observe motivational variables related to the context and self in profile B participants–the way their academic and professional backgrounds make them feel more able to apply.

#### 3.2.2. Features of the Training Model

All participants interviewed spoke positively about several aspects of the training model, especially highlighting general aspects such as the team, materials provided, and group format, noting these as significant contributions to the training.

However, when discussing specific levels of training, the distinction between the participants in the two profiles became clear. Although both mentioned Level 1 (the theoretical level), the participants in Profile A seemed to grasp the concepts better and talk more about the impact of this level on the rest of the training.

P1A: *[…] However, I think this theory is essential for us to understand. It makes perfect sense for us to only reach history at the second or third level and have the baggage that we already have from our training. Still, it was a refresher in ideas, concepts, and theories-there were even more recent theories that I did not know at all, and that was important too-and then put them into practice*.

P2A: *First, do the theoretical terms, right? […] I had a basic idea, but not so consolidated, that the stories make them mirror the character […] “ok, I also think this way”, or “I think differently”, or “I also feel this way”, or “I feel things differently”, “why?”, ‘What would I do differently?’. […] It is going to work on SR, self-determination, and decision-making […]. I’ve learned new concepts such as the construction of arguments, the type of question, and the type of questioning […] I taught narratives at university, but I have never heard of these terms. It’s all new to me. I think it makes perfect sense.*

On the other hand, those in Profile B mentioned that it helped them remember concepts that were no longer present.

P1B: *Because of this issue of recovering the basis that, in the middle of professional practice, we go into automatic mode and have to renew and recover some concepts.*

P2B: *[…] It allowed me to recall concepts and deepen others that I did not have in my practice, which were intuitive or acquired through theoretical curiosity, right? It helped me to better structure theoretical concepts so that I could apply them in a more valid and productive way.*

Profile A and Profile B participants valued the practical aspects of the training.

P2A: *And I think there was also a practical part, so we could know how to apply it and use it with our children, right?*

P2B: *I think the script you taught us to make is very important. I always had a common thread from session to session. More attention should be paid to this aspect. Do not have many objectives. Having smaller objectives. (…) I think I found it easier to structure the sessions.*

Participants in Profile B had some barriers regarding the internal aspects of the training, namely, schedules, exercises, and evaluation.

P1B: *My only issue is that I think the last two sessions could have been compiled a bit more often. […] It would help to compile those two sessions a bit more […], we probably did not need many hours. On the last day, perhaps we did not require many hours.*

P2B: *If there are more practical sessions, it would be good. I thought that the third level went by too quickly. […] I would have liked more exercise.*

P1B: *[…] The fact that we were being evaluated was strange to us. I am honest.*

In this theme, contextual variables followed the Self-System Model of Motivational Development (Dincer et al., 2019; Skinner et al., 2008) since the participants refer to aspects of the training that influenced their experience and motivation. In addition, we can also see some interaction variables between the self and context when they say, for example, that the theoretical level recalled what they had already learned.

#### 3.2.3. Dispositional Profiles

Regarding dispositional variables, all participants reported being actively involved in the training through their engagement during the sessions or preparation beforehand.

P2B: *I think it is important for a person—some people do not value it—but I think it is important for a person to read beforehand, at least listen to the video, and not come to the sessions with nothing; I think it is important.*

P2A: *I usually transcribed the sessions when I could, when I had the chance. I transcribed everything in a session. Therefore, it took me an age to transcribe everything. I also understood the content better, so that I could perform practical exercises later.*

The key difference between the two profiles on this topic is their motivations. While all participants demonstrated motivation, those in profile A expressed motivation primarily to participate in the training:

P1A: *[…] Because there was so much enthusiasm and the ideas were all in our heads, and then during the session, we would say, “No, let us do it now, it flows much more easily here”, and we would do the whole activity straight away, even if it were just to do it on the day, we would already have the activity done.*

P2A: *[…] I have also listened to the theoretical part. Of course, I would like the practical part better, but I would also like to read the theoretical part.*

By contrast, participants in profile B were more motivated to apply the tool and viewed the training more instrumentally.

P2B: *I thought that it was a helpful tool. Useful and that it can be beneficial, in terms of all this training. I think this is beneficial for the department.*

In this theme, the participants mainly addressed action variables—what they did and what behaviours they adopted to reach the goal, which in this case was to learn how to apply the narrative-based intervention tool. In addition, we can also see the outcome variables: the fact that they felt ready to implement shows that the learning process occurred.

## 4. Discussion

Quantitative data showed a significant increase in declarative knowledge, which aligns with our initial hypothesis. This suggests that Level 1 of the training, focused on the theoretical content, positively reinforced participants’ knowledge by providing them with a solid base of information and concepts. This result is consistent with recent research on declarative knowledge development, which highlights the importance of structured educational interventions and repeated exposure to information in enhancing knowledge acquisition and retention (e.g., Speckmann & Unkelbach, 2024). Regarding procedural knowledge, an analysis of percentiles showed a transfer from declarative to procedural knowledge, as all participants achieved positive results in the task. Additionally, the interquartile range (21.25) indicated variations in performance within a heterogeneous group, showing that the exercise effectively captured participant differences. A high interquartile range suggests that while some participants may have mastered the assessed skills, others may not (Field, 2012). Therefore, although all the participants performed well, the exercise highlighted individual differences and identified the most proficient participants. The heterogeneity in procedural knowledge acquisition observed across participants may be attributable to various factors, e.g., differential levels of prior professional exposure to comparable methodologies, individual differences in cognitive processing and learning preferences, and varying degrees of familiarity with narrative-based therapeutic approaches. Psychologists with vast experience applying analogous tools in their professional contexts likely demonstrated greater competence in the simulation exercises, reflecting more developed procedural schemata. Conversely, those requiring additional scaffolding to operationalize theoretical constructs may represent a cohort that would benefit from extended skill consolidation periods. These findings underscore the pedagogical imperative for differentiated professional development programs that account for baseline variability in professional experience while ensuring equitable competency attainment through adaptive support structures.

Regarding self-efficacy, although there was a slight increase in mean scores across the three assessment points, this increase was not statistically significant. This finding contradicts our hypothesis that training improves participants’ self-efficacy. There are several potential explanations for this outcome. One possibility is the ceiling effect of this variable. It is possible that the participants, being established professionals, possessed a high level of self-efficacy in the pre-test stage, leaving little room for noticeable improvement (Tschannen-Moran & Hoy, 2001; Visoso, 2024).

Additionally, other factors, such as the participants’ initial exposure to the “The Incredibles Adventures of Anastácio, the Explorer” tool, may have influenced the absence of a significant increase in self-efficacy. The novelty of the tool may have introduced uncertainty or hesitation, counteracting the increased confidence expected from the training (Klassen & Durksen, 2014). In addition to these results, another highly relevant finding was that not all participants followed the expected pattern of performing similarly in declarative knowledge and self-efficacy; for example, higher knowledge was associated with higher self-efficacy (Boswell, 2013; Constantine et al., 2019). Some participants deviated from this pattern, with some showing patterns of more knowledge but less self-efficacy and others showing less knowledge but more self-efficacy. This contrast prompted us to delve into the fourth qualitative question to understand the reasons for these unexpected results.

The qualitative findings highlight how training is perceived by different profiles of participants, focusing on three themes: participants’ academic background and professional experience, features of the training model, and dispositional profiles. Profile A participants emphasised the importance of theory for understanding and applying new concepts, highlighting the role of theoretical knowledge in building a strong foundation for further learning. This aligns with recent research indicating that a deep understanding of underlying principles enhances the transfer and flexible application of knowledge to novel situations (Soderstrom & Bjork, 2015). Participants in profile B often referred to their professional experience as a key factor in effectively applying the story tool, showing that their experience fostered confidence in their ability to meet its requirements (Tschannen-Moran & Hoy, 2001). Participants also viewed training from an instrumental perspective, aligning with studies indicating that adults favour practical learning and find it directly applicable to their work contexts (Brown et al., 2014). These findings suggest that training programs should balance theoretical and practical components to cater to diverse needs and enhance the retention and application of knowledge.

The quantitative and qualitative results present complementary facets of the impact of the training program. Initially, the quantitative data indicated a significant increase in participants’ declarative knowledge, demonstrating the positive effect of level 1 (i.e., theoretical level) on their learning. Through interviews, we found that participants unanimously emphasised the importance of this level, either as a reinforcement of previously acquired knowledge or as a crucial foundation for applying the tool. This reinforces the consistency with existing literature (e.g., Ertmer & Newby, 2013). Regarding procedural knowledge, the interview data highlighted that all participants found creating a session script essential, as it bridged the gap between theoretical knowledge and practical experience. Interestingly, despite scoring lower in knowledge assessments, participants in profile B placed the highest value on prior training and the knowledge acquired. This may be related to the potential overestimation of the existing base knowledge.

Kornell and Bjork (2007) state that individuals often confuse familiarity or fluency with the material studied with proper understanding and retention. This can lead to a false sense of competence in which individuals overestimate their knowledge. This can result in inappropriate strategies, such as revising less than necessary or avoiding learning techniques that seem more difficult. Regarding self-efficacy, the interviews revealed that participants needed more practical exercises to feel more confident, which may help explain why the increase in self-efficacy data was not significant. This is consistent with research highlighting the central role of mastery experiences in the development of self-efficacy (Schunk & DiBenedetto, 2020), which is further evidenced by profile A’s participants, who reported lower self-efficacy despite achieving high scores in knowledge and expressing a need for more practice.

Additionally, the interviews confirmed that several general characteristics of the training model were appreciated and contributed to the participants’ motivation and engagement, helping them to understand the positive impact of the training. These factors include the materials provided, support from the training team, and the platforms used. Another significant aspect was the simulation session. The interviews indicated that participants valued the simulation session and felt it fostered their willingness to apply the SR intervention program based on the narrative “The Incredibles Adventures of Anastácio, the Explorer” in their professional lives. Future research should deepen our understanding of the dispositional and training variables contributing to the presented discrepancies. Various factors, such as individual traits and personal preferences, may have influenced the training program’s impact. Research indicates that personal factors–including individual values, interests, and outcome expectations–significantly influence motivation and engagement (e.g., Rodrigues et al., 2025; Sitzmann & Weinhardt, 2015). Future studies should explore these factors in greater depth to develop tailored and effective training programs. Nevertheless, this interaction between qualitative and quantitative findings showed that the training had a positive impact, with participants feeling knowledgeable and capable of starting to apply the intervention tool.

### 4.1. Limitations and Implications for Research

Despite its strengths, the current study has some limitations that could be considered when interpreting findings and planning future research. First, there are some acknowledged limitations: (1) It would have been beneficial to reach all eligible psychologists for this study to increase its scope and the robustness of the findings. Nonetheless, we contacted 15 rehabilitation centres, resulting in 40% participation in training; (2) Self-efficacy was assessed solely through a questionnaire, which may have led to a potential overvaluation effect. Therefore, combining this method with another approach that does not rely on self-reporting is advantageous. (3) Another limitation of this study is that one of the four participants in profile B (i.e., P1B) had prior training and experience with the narrative tool. While this may have influenced their learning process and responses, our qualitative data suggest that the training still contributed to refreshing and deepening their previous knowledge, particularly by helping the participants revisit theoretical foundations and update previous concepts. Future studies should consider stratifying participants by prior experience and training to assess the differential impact of new training programs. Some factors may have acted as limitations, although we could not measure them, such as the difference in the delivery modality between the first and second editions of the training program. Due to the geographical distance of the participants in the second edition, conducting the simulation session in person was not possible, which may have influenced their experience. Therefore, future studies should be undertaken to ensure that all participants undergo the same conditions. Additionally, the small sample size in the present study did not allow for statistical comparison between online and in-person delivery modalities. As a result, we could not determine whether the mode of delivery had a significant impact on training outcomes. Future research with a larger sample should investigate the influence of delivery modality better to understand its potential effects on the training program. Future research should recruit participants with high baseline variability and employ more granular self-efficacy scales to mitigate ceiling effects in self-efficacy measurements. The tool’s novelty may have temporarily dampened efficacy gains; thus, training adaptations—such as structured practice, peer reflection, and spaced reinforcement—could enhance familiarity and confidence. Collectively, these refinements may yield more discriminative and sustained self-efficacy improvements. Finally, the simulation session was not carried out individually using these data for statistical purposes. Not all participants performed the same tasks, and the group effect could have influenced the results. However, we believe that the development of these exercises remains positive as they provide participants with some insight into how to apply the tool in practice. Future studies should consider individual simulation sessions to isolate participants’ performance and learning outcomes better.

### 4.2. Implications for Practice

This study had two major practical implications. First, it provides experienced professionals who may sometimes be distanced from theoretical concepts and the opportunity to revisit and acquire new knowledge. It also allows them to learn how to use a tool well-tested and validated in the literature. This will enable them to update their practices and provide more opportunities to develop SR skills. Second, systematically integrating theoretical and practical elements offers a potential framework for researchers developing training interventions. The findings underscore the critical role of combining knowledge consolidation with applied learning opportunities to optimise training efficacy.

## 5. Conclusions

The data show a significant increase in declarative knowledge, confirming our initial hypotheses. As for procedural knowledge, percentile analysis indicated a transfer from declarative to procedural knowledge, with all participants performing positively on the task. Regarding self-efficacy, although there was a slight increase in the average scores of the three assessments, this increase was not statistically significant. Since not all participants followed the expected pattern and demonstrated more knowledge but less self-efficacy, this prompted us to explore the reasons behind these unexpected results further using qualitative methods. The qualitative analysis employed participant-derived profiles, constructed from knowledge and self-efficacy scores, as an exploratory framework to investigate potential variations in training perceptions. Owing to the limited sample size and heterogeneous participant demographics, these profiles were not conceived as definitive or generalisable categories but rather as heuristic tools to identify emergent patterns warranting further study. The thematic analysis revealed three salient dimensions: academic background and professional experience, the characteristics of the training model, and dispositional profiles. Participants with more knowledge and less self-efficacy tended to value theory in understanding and applying new concepts. In contrast, those with the opposite profile highlighted their professional experience and preferred practical learning. However, these observations are preliminary and should be interpreted with caution. The interviews also confirmed the importance of various aspects of the training, such as the materials provided, the support of the training team, and platforms used, and the simulation session, which encouraged participants to apply the SR intervention program based on Anastácio’s narrative in their professional practice. Overall, the current study offers preliminary empirical evidence regarding how established practitioners might enhance both their theoretical understanding and practical methodologies by applying validated assessment tools to support the development of SR skills in children with CP. Beyond that, it serves as a model for other researchers, showing that the combination of theory and practice and personalised support improves the effectiveness of training. Further research with more homogeneous samples is needed to expand and confirm these exploratory findings.

## Figures and Tables

**Table 1:** Description of the training program levels.

Level	Module	Hours	Total
1	Motivation Self-Regulation Self-Determination Gamification Engagement in rehabilitation Parenting Narrative and narrative-based intervention	20 h (16 synchronous hours + 4 asynchronous hours)	60 h (44 synchronous + 16 asynchronous)
2	“The Incredibles Adventures of Anastácio, the Explorer”: Story “The Incredibles Adventures of Anastácio, the Explorer”: Program’s structure “The Incredibles Adventures of Anastácio, the Explorer”: Program’s methodological tools	18 h (12 synchronous hours + 6 asynchronous hours)	
3	“The Incredibles Adventures of Anastácio, the Explorer”: Observation and analysis “The Incredibles Adventures of Anastácio, the Explorer”: Simulated learning	22 h (16 synchronous hours + 6 asynchronous hours)	

**Table 2:** Sociodemographic characteristics of participants.

Characteristic	*n*	%
Gender		
Man	1	5.9
Woman	16	94.1
Age		
<30	1	5.9
30–45	13	76.4
46–60	2	11.8
>60	1	5.9
Nationality		
Portuguese	17	100
Academic qualifications		
Bachelor’s degree	6	35.3
Master’s degree	11	64.7
Years of experience as a psychologist		
<7	2	11.8
7–14	7	41.2
14–25	6	35.3
>25	2	11.8
Years of experience as a psychologist in a rehabilitation context		
<7	5	29.4
7–14	8	47
14–25	2	11.8
>25	2	11.8
Previous training on the topics covered by the training program		
Yes	4	23.5
No	13	76.5

**Table 3:** Multivariate tests.

Test	Value	F	Hypothesis df	Error df	Sig.
Wilks’ Lambda	0.929	0.572	2	15	0.576

**Table 4:** Descriptive statistics.

	Mean	Std. Deviation	N
Self-efficacy pre-test	3.3765	0.41912	17
Self-efficacy post-test 1	3.4118	0.38060	17
Self-efficacy post-test 2	3.4471	0.31843	17

**Table 5:** Description of the themes.

Theme/Subtheme	Categories
Participants’ academic background and professional experience Previous training Previous experience with the tool Professional experience	Contributions Barriers
Features of the training model General Training Characteristics Level 1 Level 2 Level 3	
Dispositional profiles Engagement Motivation	

## Data Availability

The data supporting the findings of this study are available upon request from the corresponding author. The data were not publicly available because they contained information that could compromise the privacy and confidentiality of the research participants.

## Supplementary Materials

The following supporting information can be downloaded at: https://www.mdpi.com/article/10.3390/ejihpe15070120/s1, Simulation task.

## Abbreviations

The following abbreviations are used in this manuscript:

CP	Cerebral Palsy
SR	Self-Regulation
