# Supplementary material for: Promoting Self-Regulation in Children with Cerebral Palsy: A Mixed Analysis of the Impact of a Training Program for Psychologists
Source: Eur J Investig Health Psychol Educ. 2025 Jun 26;15(7):120. doi: 10.3390/ejihpe15070120 (PMC12294751; doi:10.3390/ejihpe15070120)
Supplement: Supplementary file 1 [file ejihpe-15-00120-s001.zip › ejihpe-3618860-supplementary.pdf]

Supplementary Material

Promoting Self-Regulation in Children with Cerebral Palsy: A Mixed Analysis of the Impact of a Training Program for Psychologists

André Oliveira [1,2], Inês Castro [2], Ana Guimarães [2], Sofia Vidal [2], Maria Carneiro [2], Beatriz Magalhães [3], Pedro Rosário [2], and Armanda Pereira [2,3*]

[1] Department of Psychology and Education, Faculty of Social Sciences and Humanities, University of Beira Interior, 6200-209 Covilhã, Portugal

[2] Psychology Research Center, School of Psychology, University of Minho, 4710-057 Braga, Portugal

[3] Department of Education and Psychology, School of Human and Social Sciences, University of Trás-os-Montes and Alto Douro, 5000-801 Vila Real, Portugal

Author note:

*Correspondence: Departamento de Educação e Psicologia, Escola das Ciências Humanas e Sociais, Universidade de Trás-os-Montes e Alto Douro, Quinta de Prados, 5000-801 Vila Real, Portugal; armandap@utad.pt

Copyright: © 2025 by the authors. Submitted for possible open access publication under the terms and conditions of the Creative Commons Attribution (CC BY) license (https://creativecommons.org/licenses/by/4.0/).

Simulation task

Session 1

Session outline:

- Welcome to the session
- Reading of Chapter I: *The Mysterious Box*
- Reflections
- Final message

Reading and exploration of chapter I

Objective:

- To reflect on the importance of self-regulation strategies in learning: Setting goals

Reflection on chapter I

- What happened in this chapter?
- Who are the characters and what are they like?

Copyright: © 2025 by the authors. Submitted for possible open access publication under the terms and conditions of the Creative Commons Attribution (CC BY) license (https://creativecommons.org/licenses/by/4.0/).

**Objective:** To reflect on the importance of self-regulation strategies in learning: Setting goals			
**Citação**	**Question / Expected Answer**<br>AS – Attentional stimulus<br>AC – Argument construction<br>DM – Decision-making<br>ACS – Alternative solution construction	**Type of knowledge**<br>DK – Declarative knowledge<br>PK – Procedural knowledge<br>CK – conditional knowledge	**Strategy/Area for Development**
"The magnifying glass nudged my hand towards an old tree, pointing me towards the goal."	1. **What do you think a goal is?**<br>*(Expected answer: something I really want)*<br>1.1. **How do we know that something we want is a goal?**<br>1.2. **When does something we really want become a goal?**<br>1.3. **Can you give me an example of a goal?** *(Have a solid example up your sleeve)*<br>*(AS | AC)*<br>2. **What was Anastácio's goal?** *(AS)*<br>2.1. **And did Anastácio really want to achieve that goal?** *(AS | AC)*<br>2.2. **From what moment?/When did he start to really want it?** *(AS)*<br>2.3. **Why was it a "no"? And how did that "no" turn into a "yes"?** *(AS / AC)*<br>2.4. **Let's take an example of a goal we don't really want at first (like eating more Brussels sprouts), but that we turn into something we *do* want. Ask the children to do the same kind of exercise.** *(AC)*<br>2.5. **Why is having a goal important?** *(AC)*	DK<br><br>PK<br><br>CK<br><br><br><br>DK<br><br>CK	Goal setting<br><br>Goals decomposition

Copyright: © 2025 by the authors. Submitted for possible open access publication under the terms and conditions of the Creative Commons Attribution (CC BY) license (https://creativecommons.org/licenses/by/4.0/).

	2.6. **In what situations/areas of our lives might it make sense to have goals?** *(e.g., hobbies and sport, school, friendships)*		
"It's curious how great adventures always begin with small and simple things."<br><br>"I unfolded the map and examined the routes with the magnifying glass. – Hey! What about us? Don't forget us – the voice was gentle, yet firm."	**3. Let's start with an example:**<br>*Imagine my goal is to eat a big bar of Milka chocolate.*<br>**Can I eat the whole bar all at once?** *(AC)*<br>*(Expected answer: No)*<br>3.1. **Why not?** *(AC)*<br>3.2. **So how can I reach this goal?** *(AC)*<br>*(Expected answer: by eating a little bit at a time)*<br>*4. (Refer back to the quote)*<br>**So, what was the first step Anastácio took to achieve his goal?** *(AS)*<br>*(Expected answer: receiving the box and the message)*<br>4.1. **And what were the next steps?** *(AS)*<br>*(Expected answer: he unfolded the map and examined the routes with the magnifying glass)*<br>*5. (Revisit the goals discussed earlier or ask the children to suggest new ones to break into smaller steps)*<br>5.1. **What should I do to achieve my goal?**<br>5.2. **How can we achieve long-term goals?** *(Use the children's own examples – focus on being process-oriented)(AC)*<br>5.3. **How can we break down a goal like 'passing the school year'?** *(AC)*	PK<br>CK<br><br><br><br>DK<br>PK<br><br><br><br>DK<br><br><br><br><br><br><br><br><br>PK	

Copyright: © 2025 by the authors. Submitted for possible open access publication under the terms and conditions of the Creative Commons Attribution (CC BY) license (https://creativecommons.org/licenses/by/4.0/).
